# Efficacy of Different Fluoride Therapies on Hypersensitive Carious Lesions in Primary Teeth

**DOI:** 10.3390/medicina59112042

**Published:** 2023-11-20

**Authors:** Mohamed Abudrya, Christian H. Splieth, Mhd Said Mourad, Ruth M. Santamaría

**Affiliations:** 1 Department of Preventive and Pediatric Dentistry, University Medicine of Greifswald, 17475 Greifswald, Germany; mohamed.abudrya@stud.uni-greifswald.de (M.A.); splieth@uni-greifswald.de (C.H.S.); mhd.mourad@uni-greifswald.de (M.S.M.); 2 Department of Orthodontics, University Medicine of Greifswald, 17475 Greifswald, Germany

**Keywords:** dental caries, dentin hypersensitivity, fluoride, minimally invasive dentistry, potassium iodide, silver diamine fluoride

## Abstract

*Background and Objectives:* This prospective, comparative, double-cohort study aimed to compare the efficacy of silver diamine fluoride and potassium iodide (38% SDF+KI; Riva Star^®^) with sodium fluoride varnish (5% NaF; Duraphat^®^) in hypersensitive carious lesions in primary teeth to evaluate caries arrest and hypersensitivity relief. *Materials and Methods*: This study included thirty 2–5-year-olds (mean age = 3.67 ± 1.06 years; 16 males and 14 females) who required a desensitizing treatment for hypersensitive carious defects with visible dentin. A total of 15 of the participants were consecutively allocated to treatment with 5% NaF, and they were further compared to an equal number of participants treated with 38% SDF+KI solutions (*n* = 15). The treatments were performed following clinical evaluation of caries activity using the International Caries Classification and Management System (ICCMS^TM^) and the Bjørndal criteria (score of 0–9). Parental-reported hypersensitivity was evaluated using a visual analogue scale (0–10 = no to severe pain). *Results*: Clinical variables were evaluated at baseline and three months after treatment. Thereafter, a significant decline in hypersensitivity/pain led to lower final scores in the Riva Star^®^ group (0.40 ± 1.12, *p* = 0.002) than in the Duraphat^®^ group (1.40 ± 2.20, *p* = 0.004). The caries arrest effect was significantly higher in the Riva Star^®^ group (86.7%) compared to the Duraphat^®^ group three months after treatment (13.3%, *p* < 0.001). In both groups, there were no statistically significant differences in the children’s behavior before, during, and after treatment. *Conclusions*: Ultimately, with both fluoride therapies reducing hypersensitivity/pain significantly, treatment with 38% SDF+KI was clearly more effective in caries arrest than 5% NaF varnish after a 3-month period.

## 1. Introduction

Dentin hypersensitivity is described as short, sharp pain resulting from exposed dentin in response to external stimuli. These stimuli could be thermal, evaporative, tactile, osmotic, or chemical, and they are not attributed to any other dental disease or defect [1]. Evidence of dentin hypersensitivity (DH) prevalence in children is scarce. Reviews by Shiau [2] and Splieth and Tachou [3] reported ranges between 3 to 73% and 3 to 98%, respectively, in adults, with a higher female incidence in the former study.

Despite the limited evidence of hypersensitivity prevalence in children, this is one of the most prevalent diseases in children (early childhood caries), and it is noted to be among the common causes of hypersensitivity in pediatric dentistry [4]. Dental caries can generally be rooted to several factors, such as an imbalance in the oral microbiome and dietary habits including consumption of cariogenic food, and poor oral hygiene [5]. Early childhood caries (ECCs) is defined as the early onset of caries in young children with often rapid progression, which can eventually result in complete destruction of the primary dentition. Epidemiologically, ECCs can be defined as the presence of one or more decayed (non-cavitated or cavitated lesions), missing (due to caries), or filled surfaces in any primary tooth of a child under the age of six [6].

Although there has been a notable increase in the prevalence of ECC in industrialized countries [7], the literature concerning strategies to manage hypersensitivity in this condition is limited. Generally, dentin hypersensitivity can be treated by interrupting the neural response to pain stimuli or blocking the exposed dentinal tubules [8]. Most commonly, sodium fluoride (NaF) varnish has been traditionally used for managing hypersensitivity by occluding the causative open dentinal tubules [9]; however, this has been shown to facilitate caries arrest [10].

In 2014, silver diamine fluoride (SDF) was approved by the US Food and Drug Administration as a treatment for dentinal sensitivity [11]. It is commonly used in several countries for treating dentin hypersensitivity associated with the presence of carious lesions [12]. SDF was previously used off-label for caries arrest; however, it was recently recommended in the guidelines of the American Dental Association as a caries-arresting medicament [13].

A concern to consider regarding SDF is the black staining of the arrested carious lesions, which may result in undesirable aesthetics [14]. Despite this concern, most parents preferred this option to advanced treatment methods such as general anesthesia [15]. Applying a saturated solution of potassium iodide (KI) immediately after silver diamine fluoride application has been claimed to minimize the staining of dentin caries [16]. This is probably due to the reaction of iodide ions from the KI solution with the excess silver ions from the silver diamine fluoride solution, which forms a precipitate of silver iodide. Moreover, it was shown that dentine permeability could be reduced if potassium iodide was applied after a fluoride-containing silver diamine solution, consequently reducing dentin hypersensitivity [17]. Hamama [18] and Koizumi [19] showed positive results regarding the desensitizing and caries-arresting effect using a combined silver diamine fluoride and potassium iodide agent (38% SDF+KI; Riva Star^®^). However, caries arrest was reported to be poorer when potassium iodide was combined with silver diamine fluoride solution in children with caries lesions with an ICDAS score of 3 or above. On the other hand, the combination had better odds of minimizing the staining effect than solely applying silver diamine fluoride [20].

Most of the available studies refer to the prevalence, causes, and approaches used to manage dentin hypersensitivity in adults. In contrast, the literature concerning dentin hypersensitivity in children is very limited [21,22], representing a clear evidence gap concerning dentin hypersensitivity management in children, specifically that of an underlying cariological etiology. Therefore, our study aimed to evaluate the capability of 5% sodium fluoride varnish to relieve dentin hypersensitivity pain in children and to assess its impact on arresting the active carious lesions of hypersensitive primary teeth compared to treatment with 38% silver diamine fluoride and potassium iodide. The null hypothesis was that no difference would be found at three months between the two arms for the primary outcome of relieving dentin hypersensitivity pain. Moreover, as secondary outcomes, we evaluated caries arrest and compared the treating dentists’ opinions on the procedures and the children’s behavior in both interventions.

## 2. Materials and Methods

This study followed a two-arm prospective interventional design conducted in the Preventive and Pediatric Dentistry Department of Greifswald University in the period from January 2020 to March 2021. Ethics approval was granted by the Research Ethics Committee of Greifswald University on the 8th of August 2018 (No. BB 128/18; trial registration no. NCT04804423). Written informed consent was obtained from the participants’ parent/legal guardian/next of kin to participate in the study to evaluate the effect of fluoride varnish and 38% SDF+KI in carious lesions.

### 2.1. Sample

Sample size was estimated using “G*power version 3.1” while considering the following parameters: T tests, effect size: 1, α err prob: 0.05, power (1-ß err): 0.8 and an expected mean difference of visual analog scale (VAS) values between the two comparison groups. The total sample size was found to be 28 patients. Assuming a drop-out of 10%, 30 participants were required.

### 2.2. Treating Dentists

Treatments were performed by six different dentists (four pediatric specialists and two post-graduate pediatric dentistry students), all of whom were briefed on the study protocol and received instructions on carrying out the interventions according to the manufacturer’s guidelines.

### 2.3. Participant Screening, Eligibility, and Baseline Assessment

**Figure 1:**
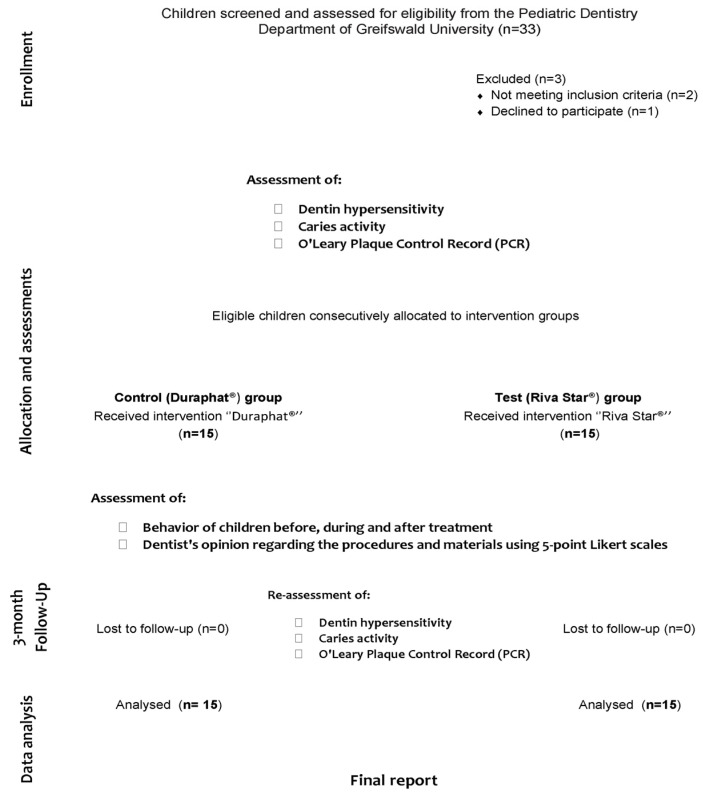
Study CONSORT diagram.

At screening, 2 calibrated dentists (kappa > 0.81) assessed, from regular clinic attendees, all eligible patients and consecutively recruited 30 healthy children who presented with active carious lesions with visible dentin (International Caries Detection and Assessment System (ICDAS) code 5) along with symptoms of hypersensitivity, and who had not used any desensitizer for at least 1 month prior to the participation date (see study diagram, Figure 1). On the other hand, children were ruled out if they presented systemic conditions requiring special dental considerations or allergies to any materials used in the study. At the tooth level, teeth that were previously restored or that clinically exhibited signs or symptoms of irreversible pulpal or periradicular pathology were excluded.

The eligibility examination comprised clinical assessment of active carious lesions according to ICCMS^TM^, Bjørndal visual–tactile criteria (Table 1) [23,24], and a reported history of hypersensitivity symptoms like short-duration sharp pain in response to thermal (cold and hot drinks or food) or tactile (toothbrushing) stimuli obtained from the parent/caregiver of the child. A hypersensitivity confirmatory test using a triple-syringe air blast on the exposed surface of the carious lesion was also used to provoke a response from the patient, allocate areas with suspected dentin hypersensitivity, and eliminate possible other causes of pain [25].

The degree of pain severity was quantified via a visual analogue scale (VAS (0–10)) [26], ranging across a continuum from none (0) to a severe amount of pain (10). Moreover, assessment of the pain magnitude was verified by the accompanying parent/caregiver, mainly due to the limited communication skills due to the age of the participants, making it puzzling to quantify pain severity from the child only [27]. Following the assessment of hypersensitivity, only 1 tooth per child was included for caries activity evaluation. Following the selection of eligible teeth, a blinded second investigator (R.M.S) randomly assigned one of the eligible teeth to be included. Children’s behavior before, during, and after treatment was assessed using Frankl’s scale (1–4 = definitely positive to definitely negative) [28].

Consequently, eligible patients were evaluated at baseline for the following:

- Hypersensitivity pain;
- Carious lesion activity and pulp status;
- Behavior of children before, during, and after treatment;
- Dentist’s opinion regarding the procedures, materials, procedure duration, and child’s discomfort within the procedure in both groups using 5-point Likert scales;
- O′Leary Plaque Control Record (PCR).

### 2.4. Treatment Procedures and Assessment

Fluoride-desensitizing therapy was explained and discussed with the participants’ parents and informed consent was obtained. Thereafter, 15 participants were consecutively allocated to receive a standard fluoride-desensitizing therapy (control arm), which included the application of 5% NaF varnish (Duraphat^®^, Colgate Palmolive Ltd., Guildford, UK) on the affected teeth. A second arm was set as a comparator, where an equal number of participants were consecutively enrolled under similar criteria and treated with 38% SDF+KI (Riva Star^®^, SDI Limited, Bayswater, Australia). Parents were always present during the treatment procedures.

The application of 5% NaF varnish followed the manufacturer’s instructions: cleaned tooth/teeth, dispersion of 5–7 mm diameter drop/up to 0.25 mL of the varnish with a brush/probe/swab as aa thin layer to all affected tooth surfaces, and a recommendation not to brush the teeth or chew food for at least 1–2 h after treatment.

The silver diamine fluoride and potassium iodide agents were applied according to the manufacturer’s instructions: cleaned and dried tooth/teeth, the application of a small amount of gingival barrier, when possible, and protection of the lips with petroleum jelly/cocoa without contaminating the treatment site.

In step 1, silver diamine fluoride solution (Riva Star^®^, SDI Limited, Bayswater, Australia) was applied using the silver brush to the treatment site, followed immediately by step 2: using the green brush provided, a generous amount of the potassium iodide solution was applied to the treatment site until the creamy white precipitate turned clear. Afterwards, all protective/isolation materials used were removed, and used brushes and capsules were discarded in accordance with local regulations.

At the 3-month recall, hypersensitivity pain (VAS), carious lesion activity (Bjørndal and ICCMS^TM^ Criteria) and the O’Leary Plaque Control Record (PCR) were re-evaluated using the same baseline methods.

### 2.5. Statistical Analysis

Normality was checked for all variables using descriptive statistics, plots, and tests of normality. Means and standard deviations (SDs) were calculated for all quantitative variables, while frequencies and percentages were calculated for categorical variables. The comparison between the two study groups was performed using independent samples *t*-test for quantitative normally distributed variables (age, dt, dmft, and PCR), and Mann–Whitney U for quantitative non-normally distributed variables (mt, ft, ds, ms, fs, dmfs, percent change of PCR) and qualitative ordinal variables (lesion activity, VAS, behavior, and the dentist’s opinion about the procedure). Chi-squared and Fisher exact tests were used for comparing qualitative nominal variables between the two study groups. The comparison between the baseline and follow-up was conducted using a paired *t*-test when the variable was normally distributed, and the Wilcoxon signed rank test was used when the variable was not normally distributed. The Friedman test was used to compare the children’s behavior at 3 different time points (before, during, and after treatment). Binary logistic regression was performed to assess the effect of different factors on the lesion inactivity (according to Bjørndal and ICCMS^TM^ criteria). Odds ratio (OR) and 95% confidence intervals (CIs) were calculated. Significance was inferred at *p* < 0.05. Data were analyzed using IBM SPSS statistical software for Windows (version 25).

## 3. Results

This prospective, comparative double-cohort study included 30 healthy children (16 males, 14 females) with a mean age of 3.67 ± 1.06, and 7.73 ± 3.16 dmft. Both cohorts consisted of 15 participants, each of which with at least one tooth with reported symptoms of dentin hypersensitivity in an active carious lesion with visible dentin (ICDAS 5) that signaled the need for a desensitizing treatment. There was no significant difference between the two cohorts regarding male/female distribution (*p* = 0.57) or dmft values (*p* = 0.46). The baseline characteristics of the participants and caries profiles are presented in Table 2.

### 3.1. Hypersensitivity

**Figure 2:**
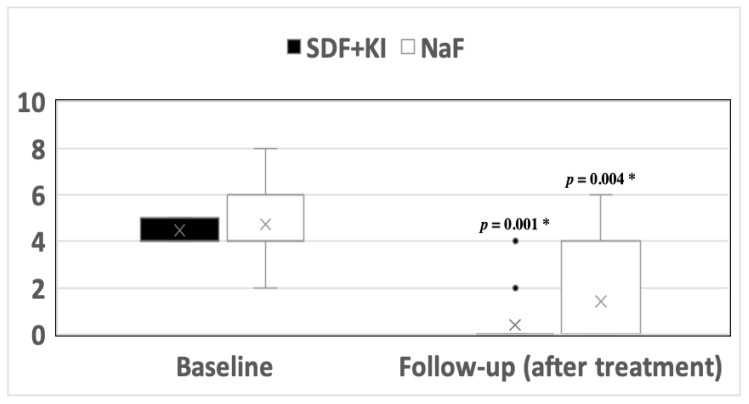
Pain assessment using the visual analogue scale (VAS) as reported by parents before and after treatment in the two study groups. SDF+KI: silver diamine fluoride + potassium iodide solution (Riva Star^®^); NaF: 5% NaF varnish (Duraphat^®^). * statistically significant at *p*-value < 0.05.

At baseline, the parents of two participants (13%) in the control arm (5% NaF; Duraphat^®^) reported severe pain (score 8, Figure 2), while moderate pain scores (4–6) dominated (*n* = 5, 33.3%, *n* = 3, 20%, and *n* = 3, 13.3%, respectively). Three months after treatment, a significant decline in pain scores (*p* = 0.004) was noticed: mostly, parents reported the absence of pain (*n* = 10, 66.7%, score 0), followed by moderate pain scores (26.6%) and mild pain (6.7%). Similarly, the assessment of pain before treatment with Riva Star^®^ (38% SDF+KI; test arm) showed moderate pain scores on the visual analogue scale (VAS, scores 4 and 5: *n* = 8, 53.3% and *n* = 7, 46.7%, respectively). After 3 months, almost all parents reported the absence of pain in their children (*n* = 13, 86.7%, score 0; *p* = 0.001). When both interventions were compared, there was no statistically significant difference in the efficacy of both treatments in relieving hypersensitivity pain (*p* = 0.31). The assessed teeth showed vitality features and no signs or symptoms of necrosis or irreversible pulpitis.

### 3.2. Caries Activity

**Figure 3:**
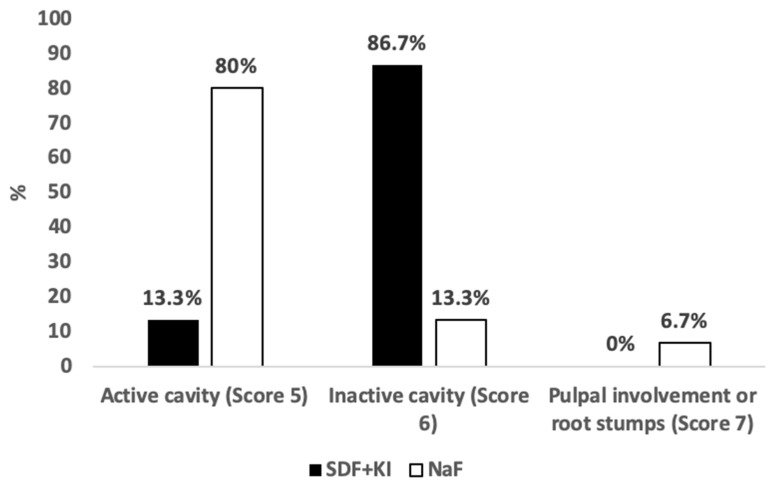
Activity of carious lesions according to Bjorndal criteria: post-operative assessment.

At baseline, all included carious lesions in the whole sample (*n* = 30) showed visual and tactile features of caries activity during examination and were graded as ICDAS 5 and as active cavities in the enamel/dentin according to Bjørndal and ICCMS^TM^ criteria. Three months after treatment with 5% NaF varnish, caries inactivation was apparent in only two (13.3%) of the treated teeth, while the remaining teeth sustained caries activity features (12; 80%), and one tooth (6.7%) manifested pulpal involvement signs. The proportion of caries arrest in teeth treated with 5% NaF varnish to those treated with 38% SDF+KI was not statistically significant (*p* = 0.10). On the other hand, the affected teeth treated with 38% SDF+KI were re-examined using the same criteria, and 13 (86.7%) out of 15 lesions were classified as inactive cavitated lesions in the enamel/dentin. Active caries were evident in only two (13.3%) lesions at the 3-month follow-up. However, the predominance of arrested lesions over the remaining active ones was statistically significant (*p* < 0.001). Comparing both treatments, the caries arrest effect was higher in teeth treated with 38% SDF+KI (*p* < 0.001, Figure 3). No signs or symptoms of irreversible pulpal deterioration were observed.

### 3.3. Children’s Behavior

In the control arm, prior to treatment, most participants exhibited positive (*n* = 6; 40%) or definitely positive (*n* = 3; 20%) behavior during the procedure (Table 3). Immediately after treatment, negative behavior was reported in two (13%) of the participants. However, differences in behavior ratings before, during, and after treatment in this arm were not statistically significant (*p* = 0.36).

Regarding the test arm, and prior to treatment, most participants showed positive (*n* = 8; 53.3%) or definitely positive (*n* = 3; 20%) behavior. Negative behavior during treatment was noticed among eight (53.3%) children, while six (40%) children equally showed a rating of either positive or definitely positive behavior. Post-operative behavior was mainly on the positive side, with six (40%) children rated as positive and four (26.7%) as definitely positive. Only two (26.7%) participants sustained negative behavior after treatment. However, significant differences were not found between behavior ratings before, during, and after treatment in the test arm (*p* = 0.07) nor between the two study arms (Table 3).

### 3.4. O’Leary Plaque Control Record (PCR)

In both cohorts (Duraphat^®^, 44.33 ± 19.35; Riva Star^®^, 37.00 ± 13.07, *p* = 0.23), the mean percentages of plaque-covered surfaces were relatively high (full mouth plaque score of 20% considered as the accepted standard) before the treatment. However, there was no significant difference when plaque percentages were re-evaluated 3 months after treatment, neither in the control (*p* = 0.72) nor in the test group (*p* = 0.32). The effect of different factors on lesion inactivation was shown in the binary logistic regression model. The percent change of plaque was inversely associated with lesion inactivity (OR = 0.99; 95% CI = 0.96–1.02). Comparing inactive lesions in both groups, there was a significant difference in mean plaque percent change (*p* = 0.04); participants with inactive lesions showed almost no change in plaque percent change in the 38% SDF+KI group (0.65 ± 38.84), while there was a noticeable percent difference in the 5% NaF group (−52.08 ± 20.62).

### 3.5. Dentist’s Opinions

Treating dentists rated the procedures as being ‘very easy’ or ‘easy’ treatments to implement (5% NaF 93.3%; 38% SDF+KI 80%). With respect to the duration of the procedures, most dentists (93.3%) considered the 5% NaF varnish application as a ‘very short’ or ‘short’ procedure to perform. On the other hand, only 66.7% of dentists classified the SDF+KI application as a ‘very short’ or ‘short’ procedure. There were no statistically significant differences between the operating dentists’ opinions of both procedures undertaken (*p* < 0.05). The distribution of the data is shown in Table 4.

## 4. Discussion

This study aimed to evaluate the effect of two fluoride therapies on hypersensitive carious lesions with visible dentin (ICDAS 5) in primary teeth. For this purpose, a comparison was set between two hypersensitivity management approaches: 5% NaF (Duraphat^®^; control arm) and 38% SDF+KI (Riva Star^®^; test arm) in terms of their potential for relieving hypersensitivity and controlling the caries activity in a sample of high-caries-risk children.

The results presented in this prospective, comparative double-cohort study comprised clinical data collected at baseline and three months later. In the literature, hypersensitivity in children is barely investigated, especially when precipitated by early childhood caries (ECC). To the best of our knowledge, this is the first study to report on clinical outcomes of this kind in this age group in Europe. However, the strict listing of indications in the only registered SDF product in Europe (Riva Star^®^, SDI Limited, Bayswater, Australia) hindered our ability to establish a randomized clinical controlled trial. Therefore, a second arm with a matching prospective design was set to be analyzed and compared with the present study data, further allowing us to establish a study with parallel intervention and control arms and to evaluate the efficacy of the existing fluoride varnish therapy with 22,600 ppm on hypersensitive carious lesions in primary teeth [9]. In addition, to avoid selection bias, parallel recruitment of both cohorts was avoided by recruiting one full cohort first and then enrolling the second cohort. We decided by chance which cohort should be recruited first. On the other hand, treatment in the present study was performed by different qualified dentists, who were briefed and trained on the use of the product according to the manufacturer’s instructions. However, only one examiner assessed the eligibility of the included subjects and evaluated the clinical outcomes at the follow-up visit. It is noteworthy to state that the evaluating examiner was calibrated on the caries classification criteria used for inclusion (ICDAS), with a substantial agreement with fellow treating dentists at a University Pediatric Dentistry department.

One of the challenges in our study was the recording and quantification of pain in children. Reliability in pre-school children’s expression of pain can be problematic, since their cognitive abilities are immature with respect to accurately remembering, reporting, or quantifying pain [29,30]. With the absence of self-report, our study utilized the responses of parents/caregivers for the assessment of pain in young children. Parental perceptions have been used in oral health surveys concerning pain assessment in preschool children [31,32]. However, parental perception of pain may differ; thus, further examination was performed when severe pain or no pain was reported to avoid the misdiagnosis of irreversible pulpitis or necrosis.

Although methods such as selective electrodes or profilometers are valuable tools for quantifying surface roughness, such as in dental erosion conditions [33], and could have provided objective data on dentinal tubule blockage and reductions in hypersensitivity, our primary focus was on assessing pain perception from the patient’s perspective and capturing the patient’s firsthand experience of, or relief from, hypersensitivity. It is worth highlighting that these objective methods could be considered in future studies to solidify our findings.

In our study, a significant decline in hypersensitivity pain intensity was observed at the 3-month mark after the application of each fluoride therapy on the affected teeth. This downturn in pain is in agreement with findings from studies that utilized comparable pain assessment methods to the Visual Analogue Scale (VAS) to assess the effect of SDF and Duraphat^®^ on hypersensitivity in adults [1,34]. These studies assessed the effects of fluoride therapies on a shorter-term basis. Therefore, our study’s data after three months prove the sustainability of the desensitizing treatments. Another study with a comparable sample size verified the short-term effects of combining silver diamine fluoride and potassium iodide for the treatment of dentinal hypersensitivity in adults [35]. These findings could be attributed to the ability of both agents to penetrate and plug the causative open dentinal tubules. With regard to SDF, silver ions are known to leave a heavy precipitate in dentinal tubules that limits permeability [36] and calcium fluoride deposits that arise from the reaction of fluoride ions with calcium ions can also block dentinal tubules [37]. Over and above, the addition of potassium iodide over silver diamine fluoride produced a precipitate silver iodide, which has been known to contribute to the blockage of dentinal tubules [17].

The sustainability of treatment with SDF+KI can be seen as a buying-time therapy until a definitive treatment, with a lower risk than treatment under general anesthesia, can be provided. However, the long-lasting effect of SDF requires an awareness of possible factors affecting the sustainability and success of caries arrest, such as attending follow-ups for monitoring caries status and the regular removal of plaque [38].

Pertaining to caries activity, the after-effect of the application of each fluoride therapy on the affected teeth in our study cannot be overlooked. The superiority of SDF+KI over sodium fluoride varnish with regard to influencing caries arrest in primary teeth is strongly supported in the literature. Despite adopting a short-term follow-up duration, our results solidify the outcomes of similar studies in this matter [14,39,40]. Therefore, we can provide evidence of the efficiency of 38% SDF+KI over 5% fluoride varnish in a timely manner. It is our belief that the reaction precipitates of silver phosphate (Ag_3_PO_4_) and calcium fluoride (CaF_2_) on the tooth level are responsible for enhancing caries inactivation, as the second precipitate, being responsible for the delivery of high doses of fluoride, resultantly enhances the chances of caries defeat. However, SDF mechanisms of action are diversely discussed in the literature [40,41,42]. Silver diamine fluoride and potassium iodide agents were used only in combination in our study. Therefore, the role of KI in caries inactivation remains unclear. In vitro studies [18,19] have shown positive results regarding the desensitizing and caries-arresting effect using combined 38% SDF+KI agents. A single study stated that SDF+KI has a higher chance of minimizing black discoloration compared with sole SDF application; however, it was also associated with a poorer carious lesions arrest rate. Yet, further clinical studies are necessary to confirm the influence of KI on caries lesions arrest [20].

On the other hand, our data revealed a potential factor impacting the probability of caries arrest following fluoride therapies. According to our analysis, the percentage change of plaque-covered surfaces is hypothesized to have an influence on the inactivation of carious lesions. However, the influence was more obvious on the inactivation of carious lesions treated with fluoride varnish than those treated with SDF+KI. Generally, disrupting the accumulation of dental plaque is known to reduce demineralizing bacterial activity and, therefore, restrain caries progression [43]. Yet, further studies with a larger sample size are necessary to affirm the influence of plaque on the inactivation of carious lesions by both fluoride therapies.

Alternately, current research has also revolved around the exploration of novel biomimetic remineralization techniques as a viable alternative to traditional fluoride-based approaches. These investigations aimed to emulate the natural mineralization process of enamel matrix and leveraging saliva-driven regeneration [44,45]. Research indicates that the use of these peptides leads to a noteworthy boost in net mineral accumulation within dental tissues [46,47]. These peptides boost mineral accumulation in dental tissues through a dual mechanism: increased mineral uptake and the prevention of mineral loss. Additionally, they stimulate the formation of new hydroxyapatite, resembling natural enamel composition [48]. However, the utilization of biomimetic nano-hydroxyapatite for enamel remineralization offers a promising alternative for preventing further demineralization at an early stage and inhibiting the onset of caries and, subsequently, hypersensitivity.

Alongside previous outcomes, our study recorded and compared the differences in children’s behavior before, during, and after treatment, as well as the operators’ views on the procedures in both groups.

Operators’ opinions were favorable for both procedures and materials used, with no statistical significance related to any of the evaluated variables. Moreover, no significant behavioral twist was found when the children’s behavior was evaluated before, during, and after treatment in both groups. Nevertheless, when dentists were specifically asked about children’s discomfort with the procedures, variable discomfort rates were recorded more in SDF+KI application procedures (73.3%) than in the fluoride varnish ones (46.7%). This could be justified by the slight metallic taste and ammonia smell of the product in comparison to fluoride varnish. Overall, our operators’ opinions on the acceptability of the SDF+KI application procedure agrees with a published article of dentists’ opinions on the matter [49].

## 5. Conclusions

The analyzed data proved the efficacy of both fluoride therapies, 5% NaF varnish (Duraphat^®^) and SDF+KI (Riva Star^®^), in managing hypersensitivity in children. Moreover, our study confirmed the superior effect of combined silver diamine fluoride and potassium iodide (Riva Star^®^) over 5% NaF varnish (Duraphat^®^) in terms of arresting carious lesions with visible dentin (ICDAS 5) after three months in primary dentition. Considering the insignificant differences regarding children’s behavior during fluoride application and dentists’ opinions of the procedures undertaken, treatment with SDF+KI can be the favored approach to manage hypersensitivity associated with active caries lesions since it is privileged with caries-arresting potential in the short run. Obtaining parental consent for the aesthetic drawback of this treatment is essential, but it should be weighed against the alternatives, which are often restricted to more risky and invasive treatments under general anesthesia for small, uncooperative children.

## Figures and Tables

**Table 1:** Bjørndal Criteria for Caries Activity and Severity Assessment [23].

Score	Definition
0	Sound
1	Active lesion in enamel, without cavity (bright surface with brown discoloration)
2	Active cavity in enamel (opaque enamel surface and loss of substance)
3	Active cavity in enamel (bright surface, brown discoloration, wet dentin)
4	Inactive cavity in enamel (bright surface, brown discoloration, and loss of substance)
5	Active cavity in enamel/dentin (yellow or light brown discoloration, wet dentin)
6	Inactive cavity in enamel/dentin (dark brown discoloration, hard and dry dentin)
7	Pulpal involvement or root stumps
8	Filled tooth
9	Missing tooth

**Table 2:** Sample characteristics.

		Test (Riva Star^®^, *n* = 15)	Control (Duraphat^®^, *n* = 15)	Total	*p*-Value
Age, mean ± SD		3.58 ± 0.95	3.76 ± 1.18	3.67 ± 1.06	0.65
Gender, *n* (%)	Males	9 (60%)	7 (46.7%)	16 (53.3%)	0.46
	Females	6 (40%)	8 (53.3%)	14 (46.7%)	
Caries index, tooth level, mean ± SD	dt	6.73 ± 2.43	6.87 ± 3.14	6.80 ± 2.56	0.90
	mt	0.13 ± 0.52	0.00 ± 0.00	0.07 ± 0.37	0.78
	ft	1.20 ± 1.86	0.53 ± 0.92	0.87 ± 1.48	0.54
	dmft	8.07 ± 2.79	7.40 ± 3.56	7.73 ± 3.16	0.57
Caries index, surface level, mean ± SD	ds	18.73 ± 10.48	14.40 ± 8.34	16.57 ± 9.56	0.25
	ms	0.67 ± 2.58	0.00 ± 0.00	0.33 ± 1.83	0.78
	fs	4.53 ± 7.61	1.87 ± 3.87	3.20 ± 6.09	0.57
	dmfs	23.93 ± 14.75	16.27 ± 8.96	20.10 ± 12.61	0.17

d = decay; missing; f = filled; t = teeth; SD = standard deviation.

**Table 3:** Behavior Using the Frankl Scale Before, During, and After Treatment in the Two Study Groups.

Characteristics		Test (Riva Star^®^, *n* = 15)	Control (Duraphat^®^, *n* = 15)	Mann–Whitney *p*-Value
Baseline	Definitely negative	1 (6.7%)	3 (20%)	0.54
	Negative	3 (20%)	3 (20%)	
	Positive	8 (53.3%)	6 (40%)	
	Definitely positive	3 (20%)	3 (20%)	
During treatment	Definitely negative	1 (6.7%)	0 (0%)	0.29
	Negative	8 (53.3%)	5 (33.3%)	
	Positive	3 (20%)	7 (46.7%)	
	Definitely positive	3 (20%)	3 (20%)	
After treatment	Definitely negative	1 (6.7%)	0 (0%)	0.62
	Negative	4 (26.7%)	2 (13.3%)	
	Positive	6 (40%)	10 (66.7%)	
	Definitely positive	4 (26.7%)	3 (20%)	
Friedman Test *p*-value		0.07	0.36	

**Table 4:** Operating Dentists’ Opinions about the Procedures Performed.

Questions		Test (Riva Star^®^)	Control (Duraphat^®^)	*p*-Value
		*n* (%)		
Procedure Undertaken	Very Easy	4 (26.7%)	9 (60%)	0.08
	Easy	8 (53.3%)	5 (33.3%)	
	Manageable	1 (6.7%)	1 (6.7%)	
	Difficult	2 (13.3%)	0 (0%)	
	Very Difficult	0 (0%)	0 (0%)	
Materials Used in this Procedure	Very Easy to Handle	9 (60%)	11 (73.3%)	0.62
	Easy to Handle	6 (40%)	3 (20%)	
	Manageable	0 (0%)	1 (6.7%)	
	Difficult to Handle	0 (0%)	0 (0%)	
	Very Difficult to Handle	0 (0%)	0 (0%)	
Procedure Duration	Very Short/Short	10 (66.7%)	14 (93.3%)	0.17
	Time Efficient	4 (26.7%)	1 (6.7%)	
	Long	1 (6.7%)	0 (0%)	
	Very Long	0 (0%)	0 (0%)	
Child’s Discomfort with the Procedure	No Apparent Discomfort	4 (26.7%)	8 (53.3%)	0.10
	Very Mild Discomfort	2 (13.3%)	2 (13.3%)	
	Mild Non-significant Discomfort	5 (33.3%)	4 (26.7%)	
	Moderate Discomfort	3 (20%)	1 (6.7%)	
	Significant Unacceptable Discomfort	1 (6.7%)	0 (0%)	

## Data Availability

All data generated or analyzed during this study are included in this article. Further enquiries can be directed to the corresponding author.
